# Portal Vein Embolization Before Liver Resection: A Systematic Review

**DOI:** 10.1007/s00270-012-0440-y

**Published:** 2012-07-18

**Authors:** K. P. van Lienden, J. W. van den Esschert, W. de Graaf, S. Bipat, J. S. Lameris, T. M. van Gulik, O. M. van Delden

**Affiliations:** 1 Department of Radiology, Academic Medical Center, Meibergdreef 9, 1105 AZ Amsterdam, The Netherlands; 2 Department of Surgery, Academic Medical Center, Amsterdam, The Netherlands

**Keywords:** Liver, Resection, Portal vein, Embolization, Regeneration, Future remnant, Colorectal metastasis

## Abstract

**Purpose:**

This is a review of literature on the indications, technique, and outcome of portal vein embolization (PVE).

**Methods:**

A systematic literature search on outcome of PVE from 1990 to 2011 was performed in Medline, Cochrane, and Embase databases.

**Results:**

Forty-four articles were selected, including 1,791 patients with a mean age of 61 ± 4.1 years. Overall technical success rate was 99.3 %. The mean hypertrophy rate of the FRL after PVE was 37.9 ± 0.1 %. In 70 patients (3.9 %), surgery was not performed because of failure of PVE (clinical success rate 96.1 %). In 51 patients (2.8 %), the hypertrophy response was insufficient to perform liver resection. In the other 17 cases, 12 did not technically succeed (0.7 %) and 7 caused a complication leading to unresectability (0.4 %). In 6.1 %, resection was cancelled because of local tumor progression after PVE. Major complications were seen in 2.5 %, and the mortality rate was 0.1 %. A head-to-head comparison shows a negative effect of liver cirrhosis on hypertrophy response. The use of n-butyl cyanoacrylate seems to have a greater effect on hypertrophy, but the difference with other embolization materials did not reach statistical significance. No difference in regeneration is seen in patients with cholestasis or chemotherapy.

**Conclusions:**

Preoperative PVE has a high technical and clinical success rate. Liver cirrhosis has a negative effect on regeneration, but cholestasis and chemotherapy do not seem to have an influence on the hypertrophy response. The use of n-butyl cyanoacrylate may result in a greater hypertrophy response compared with other embolization materials used.

## Introduction

Liver resection is in many cases the only option for long-term survival for patients with primary or secondary liver malignancies. Unfortunately, only 10–20 % of patients with colorectal liver metastases are candidates for liver resection. The resectability rate for hepatocellular carcinoma is approximately 20–30 % in patients with normal livers but is reduced in patients with cirrhotic livers [1]. In literature, the postoperative liver failure rate ranges from 0 to 30 % and is still the major cause of death following major liver resection. When patients prove unresectable because of insufficient remnant liver volume, portal vein embolization (PVE) is one of the methods to stimulate growth of the future remnant liver (FRL), thereby sustaining the possibility of extensive liver resection.

The first to demonstrate the regenerative capacity of the liver following portal vein occlusion were Rous and Larimore in the 1920s. In a rabbit model, they showed atrophy of the hepatic lobe ipsilateral to the ligated portal branches, while compensatory hypertrophy was observed in the contralateral lobe [2]. In 1961, portal vein ligation was reported in humans as part of a two-stage extended hepatectomy [3]. Kinoshita et al. [4] reported the first preoperative PVE in a human being in 1986. Since then, numerous reports have shown the efficacy of inducing compensatory hypertrophy of the FRL after PVE in preparation for surgery to resect primary or metastatic cancers in the liver [5–7].

Several techniques for portal vein occlusion have been reported, including intraoperative portal branch ligation [8–10], transileocolic PVE [11–13], and the percutaneous transhepatic ipsilateral [14, 15] or contralateral [16, 17] PVE technique. The underlying principle is to block the portal venous blood flow to the liver segments that are planned to be resected. This induces atrophy of the ipsilateral liver segments and compensatory hypertrophy of the contralateral liver segments, resulting in increase of the size of the FRL. In addition to the different techniques, different embolization materials are used clinically, e.g., polyvinyl alcohol particles (PVA), coils, gelatin sponge, n-butyl cyanoacrylate and lipiodol, or fibrin glue.

Many clinical studies have been published on the effects of PVE on hypertrophy of the FRL in small and larger patient cohorts. However, only few data have been published on the difference between the use of different embolization materials and the effect of chemotherapy or preexisting liver cirrhosis on the growth of the FRL after PVE.

In 2008, a meta-analysis was published by Abulkhir et al. which reviewed all publications on PVE between 1990 and 2005. They focused especially on the differences between various access techniques (transhepatic vs. transileocolic) regarding the ensuing hypertrophy response and surgical outcome [18]. However, with the growing availability of radiological intervention suites, during the past decades, the percutaneous transhepatic technique has become the standard technique for PVE. In addition, many new articles on PVE have been published since Abulkhir’s report.

In this review, we systematically evaluated all publications on PVE in the past 20 years to assess the technical and clinical results of PVE, with special interest in the influence of chemotherapy, preexisting liver cirrhosis, cholestasis, and the use of different embolization materials on the hypertrophy response.

## Materials and Methods

### Search Strategy

A systematic literature search was performed in Medline, Cochrane, and Embase from January 1, 1990 to May 1, 2011. The applied search heading was: “portal vein embolization” limited to clinical studies, including at least 10 cases, published in the English language. Titles and abstracts were screened to identify potentially relevant articles. Referred and related articles also were checked. Articles were selected following the selection criteria and were independently evaluated by two of the authors (vL, vdE), using a scoring list. The final selection was made in consensus.

### Selection Criteria

All clinical studies on PVE were included for further analysis. Full-text articles were retrieved and were included if they contained information on patient characteristics, indication for PVE, pre- and post-PVE liver volumes or percentages of the FRL, the technique that was used, time between PVE and CT/surgery, results, and complications of PVE, as well as results of liver surgery.

After the initial search, articles were excluded because they were written in a non-English language, were reports about portal vein ligation, were animal studies, were articles concerning chemoembolization, or were review articles. Furthermore, articles were excluded when patient characteristics, indications, methods, and results were not adequately described or when the FRL data were not sufficient and could not be extracted from the published data. Articles that overlapped with previously published data, that were published by the same author, or overlap with patient cohorts from the same study group or combined study groups was suspected were excluded.

### Study Quality and Data Extraction

All included studies were evaluated for study quality characteristics by two reviewers (vL, vdE) independently. Study quality was assessed using an adapted version of a checklist of the Dutch Cochrane Centre [19].

The main points of interest included: (1) patient characteristics (number of patients, age, sex, type of liver tumor, liver fibrosis, chemotherapy); (2) indication for PVE (minimal percentage FRL based on CT volumetry data or indocyanine green (ICG) clearance); (3) embolization technique (transileocolic, transhepatic ipsilateral, transhepatic contralateral) and embolization material used (polyvinyl alcohol particles (PVAc), gelatin sponge, n-butyl cyanoacrylate, fibrin glue, ethanol, coils, vascular plug, or a combination); (4) data on CT volumetry; (5) follow-up (PVE success rate (successful occlusion of the portal vein), clinical success rate, post-PVE complications and morbidity); and (6) surgical outcome (percentage and type of resection, postoperative complications and mortality). Articles were valid and used for data extraction if the above-mentioned points were described clearly.

## Results

The broad initial search using the search heading “portal vein embolization” resulted in 961 publications. Primary survey of the abstracts and articles excluded 684 articles dealing with subjects other than PVE, experimental animal studies or articles in a non-English language.

**Fig. 1:**
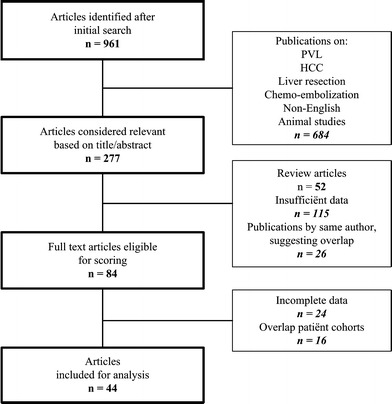
Flowchart showing selection of papers for analysis

After critical evaluation of the remaining full text articles, 84 articles remained for the final scoring, using an item-list with the minimum requirements for final inclusion. Finally, 44 publications [5, 9, 11, 12, 14–17, 20–55] were included for meta-analysis (Fig. 1), consisting of 1,791 patients, including 1,139 men (63.6 %) and 617 women (34.5 %). The sex of the remaining 35 patients could not be extracted from the articles. The mean age was 61 ± 4.1 years. The underlying pathology is summarized in Table 1.

**Table 1:** Underlying pathology

	No. of patients (%)
Colorectal metastasis	709 (39.6)
Cholangiocarcinoma	518 (28.9)
Hepatocellular carcinoma	365 (20.4)
Gallbladder carcinoma	164 (9.2)
Other (NET, angiosarcoma, cystadenocarcinoma)	32 (1.8)
Benign (adenoma, hemangioma)	5 (0.3)

### Indications for PVE

The indication for PVE varied in literature, but the percentage of the FRL was mainly used as the criterion for PVE. A resection of more than 70–75 % of the total liver volume in normal livers and more than 60–65 % in compromised livers (i.e., cirrhosis, fibrosis) was mainly the threshold for performing preoperative PVE in most studies. Three studies [20–22] used the ICG plasma disappearance rate or retention rate at 15 min [56]. A 15-min retention rate of more than 15–20 % in combination with a large liver resection constituted an indication for PVE.

In the pre-procedural workup computed tomography (CT) scans were performed to measure the volumes of the total liver, the part planned to be resected, total tumor volume, and the FRL. In most studies (30/44, 68.2 %), the absolute volumes were used to calculate the percentage FRL [5, 9, 11, 12, 14–17, 23–45, 57].

$$ \% {\text{FRL}} = \frac{\text{FRLV}}{{{\text{TLV}} - {\text{TV}}}} \times 100\% $$

In the other studies (14/44, 31.8 %), the total estimated volume (TELV) was calculated using CT volumetry in combination with the body surface area, in the equation:

${\text{TELV}} = \left( {{\text{total}}\;{\text{liver}}\;{\text{volume}} - 706.2} \right) \times {\text{body}}\;{\text{surface}}\,{\text{area}} + 2.4$ as previously described [58] or using the standardized FRL (sFRL), which was calculated by dividing FRL-V (measured by CT volumetry) by total liver volume (^cal^TL-V), which was calculated using a formula described by Vauthey et al. [59]:

$$ ^{\text{cal}} {\text{TL-V}} = - 7 9 4. 4 1+ 1 2 6 7. 2 8\times {\text{BSA}},\,{\text{with}}\,{\text{BSA}} = \sqrt {{\text{height}}\;({\text{cm}}) \times {\text{weight}}} \;({\text{kg}})/3,600 $$

### PVE Technique

PVE is performed by using a transileocolic or transhepatic approach. The transileocolic approach requires a minilaparotomy or can be performed as part of a two-stage resection. Using the transhepatic approach, the procedure can be performed by ipsilateral or contralateral puncture (Table 2).

**Table 2:** Technique of PVE

	No. of procedures (%)
*Procedural approach*	
Transileocolic	223 (12.4)
Transhepatic ipsilateral	963 (53.8)
Contralateral	605 (33.8)
*Embolized branches*	
Segment 5–8	1,430 (79.9)
Segment 4–8	209 (11.7)
Segment 1–4	41 (2.3)
Other/unknown	111 (6.2)

The embolization materials mainly used for PVE were PVA, gelatin sponge, fibrin glue, n-butyl cyanoacrylate with lipiodol, polidocanol-foam, or combinations of these materials with coils or Amplatzer vascular plugs (Table 3). Gelatin sponge and n-butyl cyanoacrylate, as the primary embolization-material, were used the most in the evaluated studies (59.5 %), often in combination with other materials.

**Table 3:** Embolization materials used

Embolization materials	No. of patients	(%)
PVA particles + coils [14, 27, 30, 47, 48, 51]	250	14.7
PVA + alcohol [25]	3	
PVA + Amplatzer vascular plug [40]	10	
Gelatin sponge + lipiodol [11, 35–37, 49, 52]	130	26.3
Gelatin sponge + coils [44, 50, 70]	71	
Gelatin sponge + thrombin + urografine [12, 33]	102	
Gelatin sponge + urografine [20, 22]	120	
Gelatin sponge + polidocanol [36]	8	
Gelatin sponge + amplatzer [45]	41	
Fibrin glue/Beriplast + lipiodol [15, 36, 39, 54]	177	9.9
N-butyl cyanoacrylate + lipiodol [5, 9, 16, 17, 24–27, 29, 31, 36, 41, 42, 47, 53, 57]	554	32.5
N-butyl cyanoacrylate + gelatin sponge [23]	11	
N-butyl cyanoacrylate + Amplatzer vascular plug [26, 42]	18	
Embol-78 [38]	51	2.8
Ethanol + lipiodol [15, 34]	159	10.2
Ethanol + gelfoam + lipiodol [43]	24	
Ethoxysclerol/air-foam [28, 32]	30	1.8
Ethibloc + lipiodol [46, 48]	33	1.8

### Success Rate of PVE Procedure and its Effect on the Hypertrophy Response

The mean time interval between PVE and the follow-up CT scan was 25.9 ± 10.1 (range, 14–42) days.

The mean technical success rate of the PVE procedures was 99.3 % (range, 86.6–100 %). Reasons for failure were the impossibility of cannulating the portal system [17, 34, 42, 46] because of altered portal anatomy caused by the tumor mass or unexpected thrombosis of the portal system due to tumor progression/invasion [17, 25, 46, 47]. The clinical success rate (successful PVE procedure, inducing enough hypertrophy of the FRL to allow resection) however was 96.1 %.

In 70 patients (3.9 %), surgery was not performed. In 51 patients (2.8 %), the hypertrophy response was insufficient to perform the resection, although the embolization procedure was successful. In the other 19 cases, 12 did not technically succeed (0.7 %) and 7 caused a complication leading to nonresectability (0.4 %). These complications consisted of severe cholangitis, large abscesses and sepsis, and portal venous or mesentericoportal venous thrombosis.

### Hypertrophy Response

The growth of the FRL as a result of PVE can be calculated or expressed in two ways:

The difference in FRL volume before and after embolization in relation to the FRL volume before embolization (percentage volume increase):

$$ \% {\text{FRL}}\;{\text{volume}}\;{\text{increase}} = \frac{{\% {\text{FRL}}_{\text{post - PVE}} - \% {\text{FRL}}_{\text{pre - PVE}} }}{{\% {\text{FRL}}_{\text{pre - PVE}} }} \times 100\% $$

The difference between the percentage FRL before and after embolization (in literature referred to degree of hypertrophy [DH]):

$$ {\text{DH}} = \% {\text{FRL}}_{\text{post - PVE}} - \% {\text{FRL}}_{\text{pre - PVE}} $$

when available, the percentage FRL volume increase was extracted from the article; otherwise, it was calculated from the available data. The mean increase of the FRL volume was 37.9 ± 0.1 % (20.5–69.4 %).

### Atrophy of the Embolized Lobe

Embolization of the liver not only causes hypertrophy of the nonembolized lobe but also atrophy of the embolized lobe. Only 10 studies, including 593 patients, contained all data on total liver volumes, FRL volumes, and the volumes of the embolized lobe before and after PVE [16, 24, 25, 34, 36, 45–49]. From these studies, we could calculate the percentage of atrophy of the embolized liver (EL), using the following equation:

$$ \% \;{\text{atrophy}} = \frac{{\% {\text{EL}}_{\text{post - PVE}} - \% {\text{EL}}_{\text{pre - PVE}} }}{{\% {\text{EL}}_{\text{pre - PVE}} }} \times 100\% $$

In these studies, the influence of the tumor volume was not taken into account. The mean percentage of atrophy of the embolized liver in these studies was −12.3 % (range, −24.5 to 0.0 %), measured 29 days after PVE (range, 14–42).

### Influence of Different Variables on the Hypertrophy Response

A meta-analysis on the variables influencing the hypertrophy response was not possible because of inhomogeneity of the studies and a limited number of articles within the subgroups. Insufficient data were available to make a strong statistical comparison between the effect of right PVE and additional embolization of segment 4 branches on the hypertrophy response. The same applies to the effect of cholestasis. For evaluation of the effect of chemotherapy and cirrhosis/fibrosis on the hypertrophy response, enough studies are available for a head-to-head comparison (Tables 4, 5). Comparing the data, chemotherapy seems to have no influence on the hypertrophy response; however, patients with preexisting chronic liver disease (cirrhosis or fibrosis) show less hypertrophy response than patients with a normal liver. Statistical significance is not given in these studies.

**Table 4:** Influence of chemotherapy on the hypertrophy response

Article	No. of patients	Chemo/non-chemo	%FRL chemo	%FRL non-chemo	Significance
Covey [14]	100	43/57	22	26	Not known
Nafidi [42]	20	13/7	45.8	41.2	NS
Ribero [51]	112	28/80	9.0 (DH)	8.5 (DH)	NS
De Baere [16]	107	97/10	56.6–71.2	83.6	NS^a^

*NS* not significant in the studies, *DH* degree of hypertrophy

^a^Significant difference in hypertrophy response was seen in patients who received chemotherapy with platin agents

**Table 5:** Influence of cirrhosis/fibrosis on the hypertrophy response

Article	No. of patients	Cirrhosis/non-cirrhosis	%FRL cirrhosis	%FRL non-cirrhosis	Significance
Cotroneo [27]	31	7/24	32.1	44.2	Not known
Farges [31]	27	14/13	24.4	41.6	Not known
Ko [38]	51	22/29	38.4	46.0	Not known
Lee [39]	29	19/10	25.4	39.4	Not known

Table 6 shows only the studies that used a single embolization material. There seems to be a trend that the use of the permanent occluding n-butyl cyanoacrylate results in a greater % FRL volume increase compared with gelatin sponge, fibrin glue, and PVA.

**Table 6:** Influence of embolization material on the hypertrophy response

Embolization material	Article	No. of patients	% Increase FRL
PVA + coils/vascular plug	Esschert [30]	10	26.1
	Libicher [40]	10	26.4
	Covey [14]	100	24.3
Gelatin sponge	Fujii [11]	30	17.8
	Imamura [33]	84	30.7
	Kakizawa [35]	14	23.8
	Kim [37]	17	27.0
	Kusaka [12]	18	21.2
	Makuuchi [20]	54	37.9
	Nanashima [49]	30	29.4
	Sugawara [22]	66	35.8
*N*-butyl cyanoacrylate	Baere [16]	107	57.8
	Barbaro [24]	26	53
	Capussotti [9]	31	48.5
	Elias [29]	68	59.1
	Giraudo [17]	146	41.7
	Sirichindakul [53]	29	27.5
	Broering [57]	17	69.4
Fibrin glue	Liem [54]	15	31.4
	Nagino [15]	105	27.4

### Complications After PVE

Fifteen articles lacked a detailed description of complications encountered after embolization [9, 11, 12, 16, 24, 31, 40–43, 49–53]. From the other 29 studies (1,179/1,248 patients), the complication rates are summarized in Table 7.

**Table 7:** Complications after PVE

	% of total patients
*Minor complications*	
Fever	36.9
Elevation of transaminase	34.8
Abdominal discomfort/pain	22.9
Nausea and vomiting	2.0
Ileus	1.2
*Major complications*	
Portal thrombosis	0.8
Embolization of nontarget vessels	0.6
Liver hematoma	0.4
Infection/abscess	0.4
Intra-abdominal bile leakage	0.3

In 0.4 %, major complications after PVE led to nonresectability of the patient. These complications consisted of severe cholangitis, large abscesses and sepsis, and portal venous or mesentericoportal venous thrombosis.

The only study to describe PVE-related mortality was published by Giraudo et al. [17]. In a group of 146 patients, 1 patient died 20 days after PVE due to lethal pulmonary embolism. No embolization material was detected in the lung. A second patient developed cholangitis and died of septic shock 39 days after PVE. All other studies reported no PVE-related mortality, resulting in an overall mortality rate of 0.1 %.

### Liver Resection

In total, 20 % (358/1,791) of the originally planned liver resections after PVE were cancelled. Seven studies (327 patients) lacked a detailed description of the cause of cancellation. In the other 37 studies (1,464 patients), 18.7 % of the planned resections were cancelled: in 6.1 % because of local intrahepatic tumor progression or newly developed metastases in the FRL, making resection impossible; in 8.1 % because of extrahepatic tumor spread (peritoneal metastases, mesenteric lymph node metastases, or lung metastases); and in 4.5 % by other causes (insufficient hypertrophy of FRL despite PVE, complications of PVE leading to nonresectability, patients refusing further treatment, preoperative mortality).

The mean period between PVE and liver surgery was 36.9 (range, 21–84) days. The types of operative procedures are summarized in Table 8. In more than 70 %, a right hemihepatectomy or extended hemihepatectomy was performed.

**Table 8:** Surgical procedures

	No. of patients	%
Right hemihepatectomy	774	43.2
Extended right hemihepatectomy	516	28.8
Left hemihepatectomy	21	1.2
Extended left hemihepatectomy	45	2.5
Trisegmentectomy right	36	2.0
Other (central resection, segmentectomy)	41	2.3
No resection	358	20.0

Complications after surgery can be divided into major and minor complications. Major complications are defined as complications that required surgical treatment and/or lead to prolonged hospital stay. Minor complications are defined as complications that could be treated conservatively, not leading to prolonged hospital stay.

In 11 publications, a detailed description of the postoperative complications after resection was lacking [9, 12, 14, 15, 25, 38–41, 46, 54]. In the other 33 articles (1,210 patients), the overall morbidity was 21.7 %. Major and minor complications are given in Table 9. The overall mortality after liver resection was 3.3 %. Primary liver failure (0.4 %) or liver failure in combination with multiple organ failure (1.2 %) caused death in 50 % of the cases. Other causes were myocardial infarction (0.1 %), sepsis (0.2 %), abdominal/liver bleeding (0.2 %), multiple organ failure (0.4 %), cholangitis (0.1 %), or unknown cause (0.4 %).

**Table 9:** Complications after surgery

Major complications	10.4 %
Liver failure	5.5 %
Portal thrombosis	0.1 %
Bile leakage	3.1 %
Abdominal/liver bleeding	1.0 %
Cholangitis	0.2 %
Myocardial infarction	0.1 %
Multiple organ failure	0.4 %
Minor complications	11.3 %
Ascites	2.6 %
Pleural effusion	2.9 %
Abscesses	1.8 %
Urine tract infection	0.9 %
Wound infection	2.0 %
Pneumonia	1.1 %

## Discussion

Since the first publication on clinical PVE by Kinoshita in 1986 [4], many articles have been published on this subject. The exact mechanisms leading to atrophy of the embolized lobe and hypertrophy of the FRL are still unknown. Recent studies have shown that in addition to the redistribution of portal blood flow PVE induces an increase in hepatic growth factor (HGF) and transforming growth factor (TGF)-α and -β, which contribute to the hypertrophy of the non-embolized lobe [60, 61].

New techniques have been developed, and new embolization materials have been used and tested. The results of PVE and its role in the management of liver malignancies is mainly based on small or larger case series; No randomized, controlled trials on the efficacy of PVE have been conducted. Only one meta-analysis has been published on PVE [18]. This meta-analysis mainly focused on the differences between the surgical transileocolic (TIPE) and the percutaneous transhepatic (PTPE) technique, demonstrating a significantly higher increase in FRL in PTPE than in TIPE. There were no differences in major complications [18]. However, with the increasing availability of radiological intervention suites, the percutaneous transhepatic technique has become the standard technique for PVE. Percutaneous PVE can be performed by an ipsilateral or contralateral approach. Using the ipsilateral approach (53.8 % of the cases in this review) has the advantage of not puncturing the healthy FRL tissue, thereby reducing the risk of complications like portal vein thrombosis, dissection, or subcapsular hematoma of the FRL. However, reverse-curved catheters or multiple lumen balloon occlusion catheters are usually necessary depending on the embolization material used. Additional embolization of the segment 4 branches often is easier when using the ipsilateral approach. The contralateral approach (33.8 %) is easier in catheterization of the right portal branches and delivering the embolization material in the direction of the portal flow. This reduces the chance of migration of embolization material in the portal branches of the FRL. This review could not extract enough data to evaluate the differences in complications of the ipsilateral or contralateral approach. However, studies by Ribero et al. [51] and Di Stefano et al. [62], which evaluated complications of the ipsilateral and contralateral approach, respectively, showed almost the same types of complications and no significant difference in complication rates.

The selection of patients for PVE is based traditionally on CT volumetry. Most studies use a FRL volume of 25–30 % of the original liver volume as threshold to select patients for PVE when no compromised liver function is expected. In patients with a compromised liver function, such as in postchemotherapy liver damage, liver cirrhosis/fibrosis, and long-lasting cholestasis, a threshold of 35–40 % is preferred as minimum FRL volume. Worldwide there is consensus on these indications. Functional information can be obtained by the ICG plasma disappearance or retention rate test at 15 min. This technique, introduced in 1980, can accurately estimate postresection remnant liver function [56]. According to the literature, only few authors, mainly Japanese, have used this method to select patients for preoperative PVE. More recently developed quantitative liver function tests, such as ^99^Tc-labelled mebrofenin hepatobiliary scintigraphy HBS [63] and ^99^Tc-galactosyl-human serum albumin (GSA) scintigraphy, could play an important role in a more accurate selection of patients for PVE.

It is important to calculate the percentage of FRL volume following PVE to ensure that enough functional liver tissue is left after resection. The importance of the size of the FRL is stressed by Ribero et al. [51]. They showed that both a small FRL and limited degree of hypertrophy (DH) are strongly associated with postoperative hepatic dysfunction. The percentage of FRL volume can be calculated by using the absolute volumes by CT volumetry or by relating FRL volume (measured by CT volumetry) to a standardized liver volume based on BSA [58, 59]. Monitoring FRL function after PVE is difficult, because only a few liver function tests can measure the specific increase of the FRL. ^99^Tc-labelled mebrofenin HBS with single photon emission tomography (SPECT) [63] and ^99^Tc-GSA scintigraphy can be used for this purpose [50, 64, 65]. De Graaf et al. showed that the increase of FRL function exceeded the increase of FRL volume, suggesting that the necessary waiting time until resection could be shorter than indicated by volumetric parameters only.

There is no consensus regarding the optimal waiting time between PVE and liver resection. We found a wide range of time intervals between PVE and the follow-up CT scan: 14–42 days (mean, 25.9 ± 10.1 days). A longer time interval allows extra growth of the FRL. However, volumetric data presented by Ribero et al. [51] show that after the initial hypertrophy in the first 3 weeks, a plateau phase is reached. This is confirmed in the study by Nagino et al. [15].

Additionally, there is the issue of induction of tumor growth by PVE. Clinical studies demonstrate that tumor progression after PVE is possible in both the embolized and nonembolized liver segments. However, so far, accurate data regarding the risk of tumor progression after PVE are currently not available [66]. In this study 6.1 % of planned liver resections are cancelled because of local intrahepatic tumor progression after PVE. This can be regarded as complication of the treatment, causing irresectability. A direct causality seems obvious and is described in literature but is not yet proven. An additional 8.1 % of the resections are cancelled because of extrahepatic tumor spread (peritoneal metastases and distant metastases). To restrict tumor growth, the time between PVE and liver resection should be limited. Furthermore, sequential transarterial chemoembolization and PVE can be performed, particularly in patients with HCC [67] to limit tumor growth.

Post-PVE chemotherapy is another option in patients with CRM. Beal et al. reported a reduction in tumor size in six of the ten patients who had chemotherapy compared with tumor growth in four of the five patients without chemotherapy. However, they also observed less hypertrophy of the FRL in patients who received chemotherapy in the weeks between PVE and resection [25]. Other studies showed no significant difference in hypertrophy response or in postoperative complications when chemotherapy was continued [68]. A few large studies evaluated in this review show no significant difference in increase of the FRL volume after PVE in patients who previously did or did not receive chemotherapy [14, 16, 42, 51]. However, de Baere et al. described a significant lower hypertrophy response in patients who received chemotherapy with platin agents. Restricted by the limited number of articles and their inhomogeneity, evaluation was only possible by head-to-head comparison.

The same applies to the effect of preexisting liver damage (liver cirrhosis and fibrosis) on hypertrophy response after PVE. Comparison of relevant studies show an impaired hypertrophy response compared with normal livers; however, statistical significance has not been demonstrated. Farges et al. [31] stated that patients with cirrhotic livers and a normal hypertrophy response had less postoperative complications. On the other hand, failure of increase of the FRL could be considered an indicator of inability of regeneration of liver parenchyma and liver resection should be avoided.

Many different embolization materials have been applied for PVE. The combination of n-butyl cyanoacrylate and lipiodol and the combination of PVA particles with coils are mostly used. Both are nonabsorbable materials, which lead to persistent occlusion of the portal branches, preventing peripheral recanalization. Because gelatin sponge is absorbable, portal recanalization is frequently seen, sometimes 2 weeks after PVE [6, 69]. PVA particles are easy to use and provide permanent occlusion in the periphery of the portal venous system. Little inflammatory reaction of the liver tissue is seen when using PVA. The use of n-butyl cyanoacrylate requires more experience of the radiologist, because delivery must be very precise to prevent embolization of nontargeted branches. Using the appropriate delivering catheters, procedure time can be decreased. N-butyl cyanoacrylate induces a strong inflammatory reaction, rendering surgical resection sometimes technically more difficult [6]. Large clinical studies that compare the effect of different embolization materials on the hypertrophy response are lacking. The data in this review suggest that the use of n-butyl cyanoacrylate results in a higher % FRL volume increase.

Finally, both the overall technical success of PVE (99.3 %) and clinical success rate (96.1 %) of PVE are very high. Only 2.8 % of the patients could not undergo a liver resection because of insufficient hypertrophy. Suggested reasons for insufficient hypertrophy after successful PVE are recanalization of the embolized portal branches, activation of underlying liver disease, and the presence of major portal hypertension with portosystemic shunting [31]. Only 0.4 % of patients appear unresectable because of PVE-related complications, such as a large subcapsular hematoma, portal thrombosis, or biliary or infectious complications in the FRL after a contralateral procedure. Overall complication rates are higher, but these complications rarely need treatment and they rarely lead to unresectability.

## Conclusions

Preoperative PVE is an effective method to increase FRL volume with a high technical and clinical success rate. The complication rate is low, but local tumor progression after PVE is an imminent cause of unresectablilty. Preexisting liver damage due to cirrhosis seems to have a negative effect on the hypertrophy response. Chemotherapy however does not seem to have any influence on the hypertrophy response, except for platin agents. The use of n-butyl cyanoacrylate may result in a greater hypertrophy response compared with the other embolization materials used.
